# Observations on the Treatment of Scratches, Mange, Etc.

**Published:** 1902-09

**Authors:** G. M. Boon

**Affiliations:** Southwick, Mass.


### REPORTS OF CASES.

### OBSERVATIONS ON THE TREATMENT OF SCRATCHES, MANGE, ETC.

By G. M. Boon, V.S.,
SOUTHWICK, MASS.

Bay mare, nine years old, had had scratches for several years, until it became a chronic condition. The owner claimed that everything had been tried in vain, and several veterinarians, after treating her for some time, gave her up as incurable. July 13 the owner called at my office with her. At that time the disease had spread up the legs to the tarsometatarsal articulation, and was very severe, with the characteristic oily discharge over the leg (the near hind), desquamation of the epidermis, and some spots of ulceration. To say the least, the prognosis looked very unfavorable, but I decided to treat her, and made up the following liniment: Epicarin and glycerin, each 2 drachms; wood alcohol, 2 ounces. To be applied once daily with a soft brush, after thoroughly cleansing.

Internally I ordered arsenious acid, 72 grains. M., div., pulv. No. 24. Sig. One powder three times daily. Potassi nitras, 8 ounces. Sig. Two teaspoonfuls three times daily in drinking water.

July 17th the owner drove the horse to my office, and I was much surprised to find a great improvement. The characteristic oily discharge had completely disappeared, the epidermis was quite dry, the ulcers had healed, and the swelling was gone. I ordered the same treatment continued. August 10 the owner called again, and I hardly knew the horse, owing to the marked improvement. The secretions were entirely checked, the hair was growing nicely, and there was no swelling of the leg; in fact, everything was in a perfectly healthy condition. I prescribed arsenious acid internally, in 3-grain doses, morning and evening, for several weeks.

At this time the owner brought a well-bred cocker spaniel puppy with him, about which he and his family were terribly upset, as they thought he had been poisoned. Its mouth, nose, eyes, etc., were terribly swollen and covered with pustules, which a low-power microscope showed to contain acarus demodex folliculorum. I informed the owner that it was a case of follicular mange, and gave a somewhat guarded prognosis. As he desired me to treat it, I made up a liniment identical with the one used for his horse, and after opening all the pustules with a fine bistoury, applied the liniment with a soft brush, and ordered him to do likewise every day for three days; then every other day until he called again, August 17th. At this time the dog was greatly improved and continued to improve, making a complete recovery in two weeks.

Fox terrier, three years old. Diagnosis: Sarcoptic mange, well developed, which I verified by finding the acarus sarcoptis communis with the aid of the microscope. The treatment in this case was identical with that used in the foregoing cases, with complete recovery in two weeks.

From my observations epicarin seems to act as a powerful astringent and alterative in such conditions of chronic scratches or grease, while in mange it is a specific, destroying the parasites (acarus sarcoptis communis, and sarcoptis demodex folliculorum), and quickly healing the lesions produced by them.

The success I have had with the remedy has prompted me to write the above for the benefit of fellow-members of the profession who have not yet tried this treatment. By its use in mange we will avoid incurring the ill feeling of clients, who, after spending their money, often find that their dog is no better, and, becoming disgusted, sacrifice the life of their most valued animal friend.

Since the writing of this article I have treated several cases of mange in cattle and three in sheep successfully with the same liniment.

---

The will of Madame Claire Louise Beauclaire, a rich widow of Toms, leaves about $2000 toward the establishment of a home for ownerless cats. Madame Beauclaire had a number of pet cats, and these are to have the best accommodations in the home she would have started.

"Collecting dogs" are just now popular in England for gathering money for charitable purposes. The Royal Berks Hospital has recently been enriched to the extent of nearly $50 in 2574 coins, which Prince, a fox terrier, collected at Wokingham. Prince is the property of a local public house keeper, whose customers amuse themselves by hiding a coin, which the intelligent terrier speedily finds, when it is transferred to a box, where it remains until the times comes for the donations to be handed over to the hospital's treasurer. It is said that a collecting dog at Paddington railway station in London has during the service collected over $3750 for charity, and still continues his good work.

